# Navigating the challenge – endovascular embolization of a giant bronchial artery aneurysm with a short, irregular neck: A case report

**DOI:** 10.1097/MD.0000000000046945

**Published:** 2026-01-16

**Authors:** Jiamin Wang, Xiaogang Hu, Hongpeng Li, Jun Lu, Ling Wang, Sen Jiang

**Affiliations:** a Department of Interventional Radiology, Jinhua Hospital of Zhejiang University School of Medicine, Jinhua City, Zhejiang, China; b Department of Ultrasonography, Jinhua Hospital of Zhejiang University School of Medicine, Jinhua City, Zhejiang, China; c Department of Radiology, Shanghai Pulmonary Hospital, School of Medicine, Tongji University, Shanghai, China.

**Keywords:** bronchial artery aneurysm, case report, embolization, tortuous and short aneurysm neck

## Abstract

**Rationale:**

Bronchial artery aneurysm (BAA) is a rare condition, but it can be life-threatening if the aneurysm ruptures. While endovascular therapy is the primary treatment, the selection of embolic material is limited by the tortuous and short aneurysm neck.

**Patient concerns:**

We report a huge, suspected ruptured BAA was unexpectedly detected in a 69-year-old man during a routine medical examination.

**Diagnoses:**

Enhanced chest computed tomography angiography and selective bronchial arteriography both revealed that the BAA was located at the proximal portion of the left bronchial artery It was characterized by a large aneurysm sac and a short, irregularly dilated aneurysm neck.

**Interventions:**

We successfully occluded the huge BAA using a single amplatzer vascular plug.

**Outcomes:**

The 6-month computed tomography angiography follow-up scan showed no signs of aneurysm recurrence.

**Lessons:**

Transcatheter embolization utilizing a single amplatzer vascular plug has proven to be an effective and cost-efficient treatment for BAAs that are characterized by a large aneurysm sac and a short, tortuous, irregularly dilated aneurysm neck.

## 1. Introduction

The bronchial artery aneurysm (BAA) is an extremely rare true aneurysm that originates from the aorta.^[1,2]^ While most BAAs are asymptomatic, rupture of BAA can result in fatal hemorrhage, including massive hemoptysis, acute chest pain, and hemomediastinum.^[1,3]^ Therefore, previous studies have recommended that the BAA should be treated once detected.^[4,5]^ Owing to its minimally invasive nature and proven effectiveness, endovascular treatment has emerged as the first-line therapeutic strategy for BAA.^[6–8]^ However, the short and tortuous neck between the BAA and aorta poses significant challenges in most cases.^[9–12]^ Additionally, embolization is complicated by distal or ectopic migration of materials, which can be attributed to the high blood flow velocity and the markedly enlarged aneurysm sac. Several cases have reported successful embolization using combined stent-graft placement and transcatheter coil embolization.^[4,7,9–12]^ In the present report, we presented a giant BAA with a short neck, and it was successfully embolized with 1 single amplatzer vascular plug (AVP).

## 2. Case presentation

This retrospective study has been approved by the ethics committee of Shanghai Pulmonary Hospital, and the ethics committee of Shanghai Pulmonary Hospital (K24-522) declared the informed consent can be waived because of its retrospective nature. The patient provided written informed consent for the publication of his medical data.

**Figure 1.**
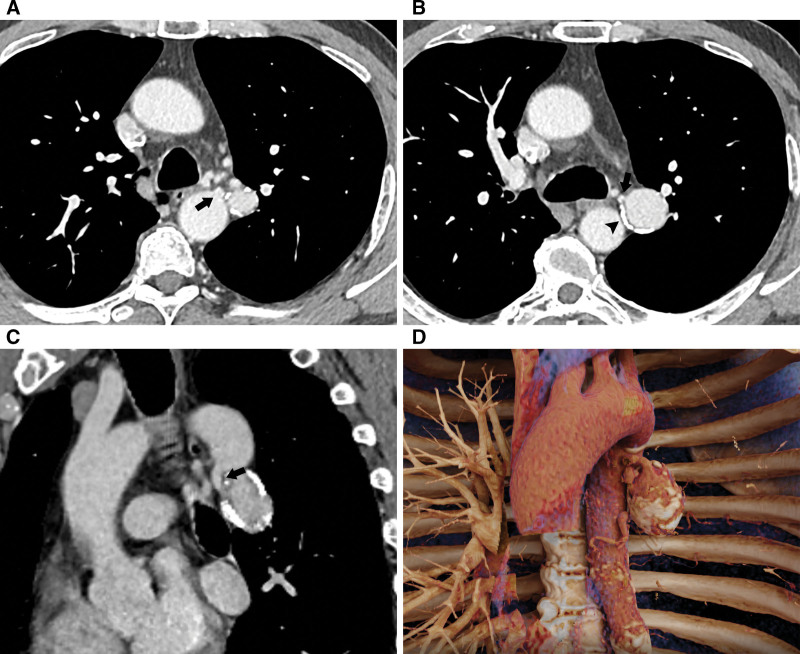
(A) A single enlarged bronchial artery aneurysm (BAA) has been identified in the left hilar region, originating from the thoracic aorta (black arrow). (B) Chest CT angiography (CTA) showed the BAA was surrounded by annular calcification (black arrowhead). Additionally, the neck of the aneurysm, adjacent to the aneurysmal sac, appeared relatively narrow (black arrow). (C) The oblique coronal chest CTA (left anterior oblique angle of 30°) distinctly revealed a short, funnel-shaped dilated aneurysm neck. (D) The 3D-VR image clearly showed the location and size the detected BAA. BAA = bronchial artery aneurysm, CTA = computed tomography angiography, VR = three-dimensional virtual reality.

A 69-year-old man presented for a routine medical examination with no obvious symptoms. Three months prior, he had experienced transient mild chest pain. Additionally, the patient had no history of chest trauma, thoracic surgery, or lung infections. He also had no history of bronchiectasis, hypertension, and coronary artery disease. Initial chest computed tomography (CT) scan showed 1 enlarged mass surrounded by annular calcification located at left hilum. Subsequent enhanced CT scans further confirmed that it was 1 single huge BAA originating from the thoracic aorta. Three-dimensional reconstruction images revealed the aneurysm originated from the most proximal portion of the left bronchial artery (BA). The aneurysm neck formed a sharp angle with the thoracic aorta and was characterized by its short length and irregular dilation. The aneurysm neck had a diameter of 6.4 mm at its junction with the aorta, and 3.4 mm at its junction with the aneurysmal sac. The length of the aneurysm neck was measured to be 14.4 mm. The longest diameter of the aneurysmal sac was 40.4 mm (Fig. 1). Severe stenosis of the basal trunk of the left lower lobe pulmonary artery, nearing complete occlusion.

**Figure 2.**
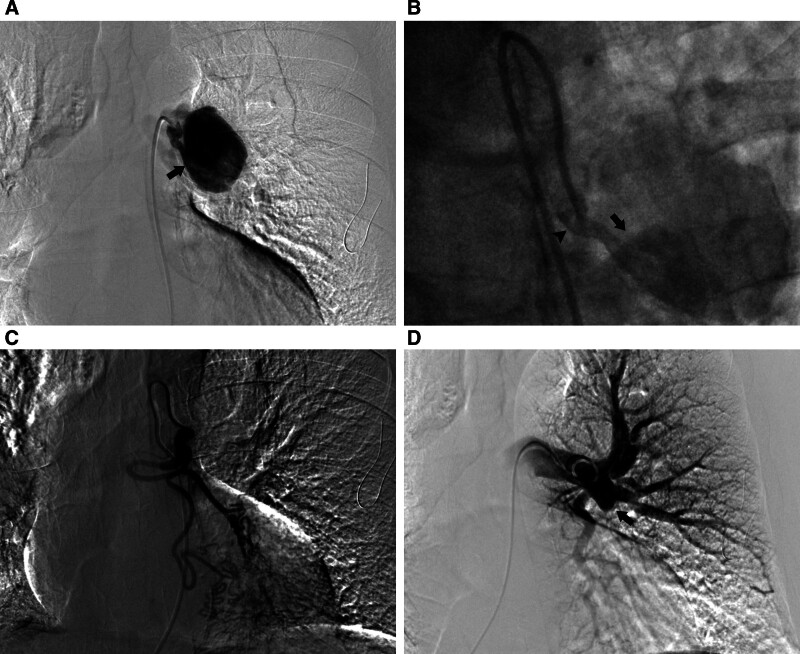
(A) Bronchial arteriography delineated the enlarged aneurysm sac (black arrow), as well as the irregularly shaped aneurysm neck; (B) the selected arteriography clearly demonstrated the elevated blood flow velocity, as indicated by the black arrow, while also revealing that the aneurysm neck had a funnel-shaped structure (black arrowhead); (C) there were no communications between the aneurysm and the left lower bronchial artery; and (D) pulmonary angiography demonstrated complete occlusion of the left basal trunk of the pulmonary artery.

The endovascular treatment was performed under local anesthesia. Firstly, selective angiography was conducted via a right femoral artery approach to confirm the location of the BAA. The bronchial arteriography images revealed left superior BA was enlarged and tortuous, and the BAA was indeed located at the most proximal portion of the left superior BA. The aneurysm neck exhibited an irregularly dilated lumen, featuring a wider proximal portion and a narrower distal portion. The funnel-shaped structure led to an increased blood flow velocity at the junction with the aneurysmal sac. The aneurysm sac was also markedly enlarged. The left lower BA had no communications with the BAA. Pulmonary angiography revealed complete occlusion of the left basal trunk of the pulmonary artery (Fig. 2).

**Figure 3.**
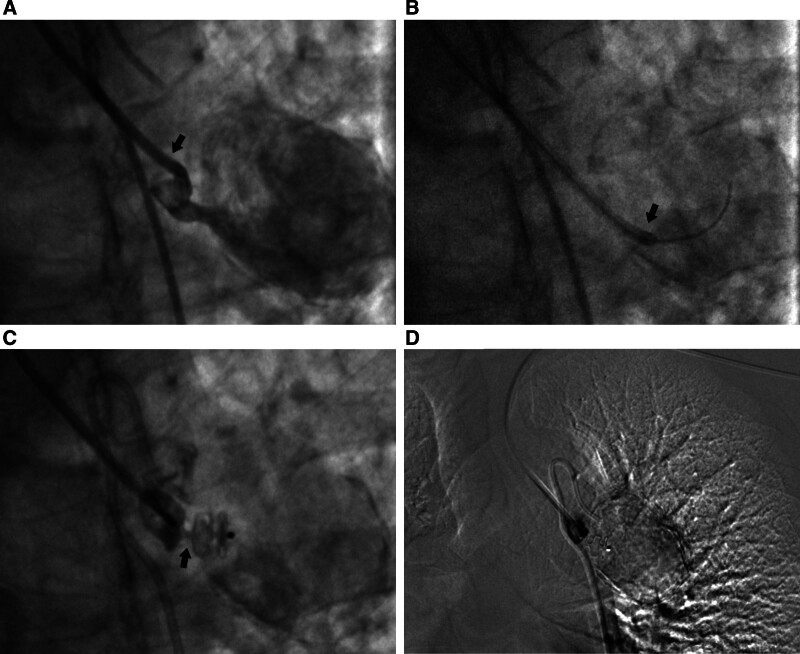
(A) One 6F MPA guiding catheter (black arrow) was introduced through the left radial artery approach; (B) the MPA catheter was positioned to anchor at the proximal portion of the aneurysm sac; (C) an AVP (length, 7 mm; diameter, 8 mm) was deployed to perform embolization (black arrow), and (D) postoperative bronchial arteriography showed the aneurysm sac was no longer visualized. AVP = amplatzer vascular plug, MPA = multipurpose angiographic.

Subsequently, selective embolization was performed via the left radial artery approach. A 6F multipurpose angiographic guiding catheter (Cordis Corp, Florida) was introduced and precisely positioned at the proximal portion of the aneurysm sac for selective catheterization. An AVP (Abbott Medical, Plymouth) was then deployed through the 6F multipurpose angiographic for BAA embolization. The distal and middle disc of the AVP (length, 7 mm; diameter, 8 mm) were both released inside the sac. The AVP was subsequently pulled back to occlude the inflow tract of the sac. The proximal disc was carefully and slowly released within the neck of the aneurysm. Finally, the postoperative angiogram confirmed that the BAA was not visualized (Fig. 3).

**Figure 4.**
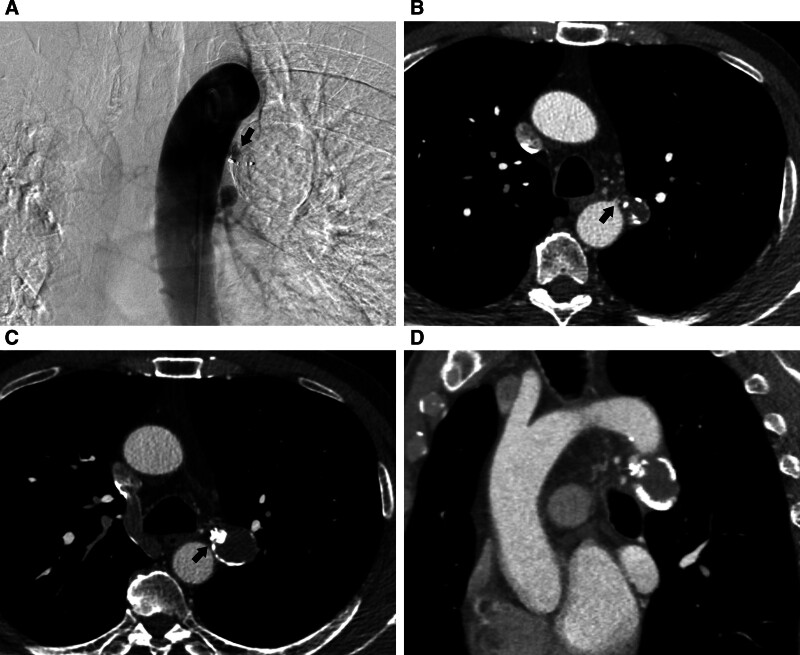
(A) Aortic angiography revealed no signs of aneurysm recurrence 1-month after embolization; (B, C) the CTA performed 6 months after embolization showed a reduction in the size of the aneurysm neck (black arrow) and no signs of aneurysm recurrence (black arrow); and he oblique coronal chest CTA also revealed no signs of aneurysm recurrence. CTA = computed tomography angiography.

The patient received only 1 day of oxygen therapy following the treatment and experienced no discomfort after the embolization procedure. The 1-month aortic angiography showed no signs of aneurysm recurrence. The patient was then followed up for 6 months, and computed tomography angiography images showed no recanalization of the occluded BAA (Fig. 4).

## 3. Discussion

The incidence of BAA is relatively low, and it is occasionally found during bronchial arteriography or enhanced CT scan. The pathogenesis of BAA remains unknown.^[2,3]^ The potential causes are identified as a consequence of increased bronchial arterial flow caused by recurrent inflammation, including bronchiectasis, chronic lung infection, and tuberculosis.^[2,4,9]^ Other uncommon causes include atherosclerosis and arteritis.^[3]^ However, about half of BAA was defined as the idiopathic aneurysm, with a clean medical history.^[4]^ In our study, no evidence of lung infection or bronchiectasis was observed. However, pulmonary angiography demonstrated complete occlusion of the left basal trunk of the pulmonary artery. We hypothesized that the detected BAA may represent a compensatory response due to the systemic-pulmonary arterial shunting.

Most of BAAs are asymptomatic. Previous studies also summarized that clinical presentation depended on whether the aneurysm has ruptured. The frequent clinical appearances caused by BAA rupture includes hemoptysis, hemomediastinum, acute chest pain, and back or epigastric pain.^[13]^ Some cases also reported that mass effect of BAA may lead to dysphagia, dyspnea, cough, or chest discomfort.^[1,14]^ Because of the high blood flow velocity of the dilated BA, acute BAA rupture has the high mortality.^[15]^ In the presented case, the patient exhibited no symptoms at presentation, but the annular calcification around BAA might indicate previously undetected self-limiting rupture. Some reports have suggested self-limiting BAA rupture may be associated with transient increased intrathoracic pressure and sudden sustained hypertension.^[16]^ The presented patient has once experienced transient emotional fluctuations which may result in transient increased intrathoracic pressure and sudden sustained hypertension.^[17]^

Considering the high mortality rate associated with BAA rupture, it is recommended that BAA should be treated as it is detected.^[5,8,9]^ In addition, previous studies have also indicated that there is no correlation between the risk of aneurysm rupture and the size of the aneurysm.^[4,5,7]^ For mediastinal BAAs, both video-assisted thoracoscope surgery and conventional surgery can achieve BAA ligation and resection, but they often entail considerable trauma.^[5]^ Therefore, endovascular treatment is increasingly becoming the first-line therapy for BAA due to its minimal invasiveness, rapid recovery, and excellent therapeutic outcomes.^[8]^ The available embolic materials include coils,^[18]^ AVP,^[12]^ particle embolic agents,^[19]^ *N*-butyl 2-cyanoacrylate (NBCA),^[20]^ and stent-graft.^[7,17]^ The location, size, and morphology of an aneurysm are all crucial factors in material selection.^[21]^

In the present study, the aneurysm was situated at the proximal segment of the left superior branchial artery. Firstly, due to the risk of unpredictable reflux and nontarget embolization, both particulate embolic agents and NBCA were deemed unsuitable. Furthermore, given the large size of the aneurysm sac, neither of the 2 embolic materials was able to achieve complete occlusion of the BAA. Secondly, we observed a high blood flow velocity within the aneurysm neck, making the use of pushable coils unsuitable due to the high likelihood of distal migration. The funnel-shaped structure of the aneurysm neck also failed to provide sufficient space for the safe anchoring of detachable coils. Previous cases have demonstrated the efficacy of combining stent-graft placement with coil embolization in similar situations.^[7,10,17,22]^ However, we did not choose this combined therapy due to the underlying risk of paraplegia and the relatively high medical cost.^[23,24]^ Eventually, we selected the type II AVP to occlude the detected BAA.

The AVP is a self-expanding cylindrical device made of a flexible nickel-titanium alloy mesh, which can be adjusted according to the shape of the blood vessel.^[25,26]^ Therefore, it can perfectly fit the funnel-shaped aneurysm neck, achieving complete occlusion in the presented case. The AVP is a detachable device with a low risk of ectopic embolism. If the deployment location is unsatisfactory, it can be easily retrieved and redeployed. The type II AVP is characterized by its 3-segment design, and its multi-layered meshes enable rapid occlusion even in high-flow vessels. As a result, we believed the AVP could offer a single-device solution, making it possible to occlude the large BAA with 1 single vascular plug. Considering the sharp angle between the aneurysm’s neck and the aorta, the left radial artery approach was more stable for delivering the AVP compared to the femoral artery approach. We deployed the distal and middle discs within the aneurysm, and both the inflated discs could achieve completely inflow occlusion of the aneurysm sac. The proximal disc which was released within the aneurysm neck provided a strong anchor to prevent distal migration.

We did not perform distal embolization of the efferent branches to avoid retrograde filling of the aneurysm, which deviates from the previous reports.^[3,10]^ In the presented case, we did not observe any communications between the other BA and the aneurysm. Additionally, the large aneurysm sac made it difficult to catheterize into the distal branch of the left upper BA. Some reports have also demonstrated the effectiveness of using a single stent-graft to occlude the BAA, which is another method of inflow tract occlusion.^[10]^ In our report, the follow-up examination result after 6 months showed no signs of recurrence. Therefore, we considered that embolizing the proximal inflow tract could also effectively occlude a large mediastinal BAA which has no communications with other branches.

## 4. Conclusion

In the endovascular treatment of BAA, a large aneurysm sac with a short and irregularly shaped neck presents a considerable challenge. We report a case of a large, suspected ruptured BAA featuring a short, funnel-shaped neck, which was successfully embolized using a single AVP. In this scenario, AVP proved to be a more feasible, effective, and cost-effective option compared to other combined therapies.

## Acknowledgments

We thank Angela Morben, DVM, ELS, from Liwen Bianji, Edanz Editing China (www.liwenbianji.cn/ac), for editing the English text of a draft of this manuscript.

## Author contributions

**Writing – original draft:** Jiamin Wang, Sen Jiang.

**Writing – review & editing:** Xiaogang Hu, Hongpeng Li, Jun Lu, Ling Wang.
